# Using Co-Design to Develop a Collective Leadership Intervention for Healthcare Teams to Improve Safety Culture

**DOI:** 10.3390/ijerph15061182

**Published:** 2018-06-05

**Authors:** Marie E. Ward, Aoife De Brún, Deirdre Beirne, Clare Conway, Una Cunningham, Alan English, John Fitzsimons, Eileen Furlong, Yvonne Kane, Alan Kelly, Sinéad McDonnell, Sinead McGinley, Brenda Monaghan, Ann Myler, Emer Nolan, Róisín O’Donovan, Marie O’Shea, Arwa Shuhaiber, Eilish McAuliffe

**Affiliations:** 1 School of Nursing, Midwifery and Health Systems, University College Dublin, Belfield, Dublin 4, Ireland; aoife.debrun@ucd.ie (A.D.B.); eileen.furlong@ucd.ie (E.F.); mcginleysinead@gmail.com (S.M.); roisin.o-donovan@ucdconnect.ie (R.O.); marie.oshea@ucd.ie (M.O.); eilish.mcauliffe@ucd.ie (E.M.); 2 Integrated Care Team Older Persons, Community Healthcare Organisation 6, Clonskeagh, Dublin 6, Ireland; deirdrebeirne@gmail.com (D.B.); sinead.mcdonnell4@hse.ie (S.M.); emer.nolan2@hse.ie (E.N.); 3 Midlands Regional Hospital Mullingar, Longford Road, Mullingar, Co. Westmeath, Ireland; ClaireConway1@hse.ie (C.C.); yvonne.kane@hse.ie (Y.K.); 4 Mater Misericordiae University Hospital, Eccles St, Dublin 7, Ireland; ucunningham@mater.ie (U.C.); akelly1@mater.ie (A.K.); amyler@mater.ie (A.M.); 5 Patient Representative, Dublin, Ireland; alanenglish6@gmail.com; 6 Quality Improvement Division HSE & Our Lady of Lourdes Hospital, Drogheda, Co. Louth, Ireland; fitzsimonsQID@gmail.com; 7 Our Lady’s Hospital, Navan, Co. Meath, Ireland; brenda.monaghan@hse.ie; 8 School of Medicine, Trinity College Dublin, Dublin 2, Ireland; ashuhaib@tcd.ie

**Keywords:** co-design, co-production, collective leadership, team performance, safety culture

## Abstract

While co-design methods are becoming more popular in healthcare; there is a gap within the peer-reviewed literature on *how* to do co-design in practice. This paper addresses this gap by delineating the approach taken in the co-design of a collective leadership intervention to improve healthcare team performance and patient safety culture. Over the course of six workshops healthcare staff, patient representatives and advocates, and health systems researchers collaboratively co-designed the intervention. The inputs to the process, exercises and activities that took place during the workshops and the outputs of the workshops are described. The co-design method, while challenging at times, had many benefits including grounding the intervention in the real-world experiences of healthcare teams. Implications of the method for health systems research are discussed.

## 1. Introduction

There has been a shift in the manufacturing industry since the 1970s to move from designing products for people (supplier-centred design), to designing them with people’s needs in mind (user-centred design), to more recently designers, suppliers and consumers coming together to look at a problem and design a solution together (co-design). The benefits of adopting co-design principles in healthcare were outlined by several authors [1,2,3] and models were proposed to identify key stakeholders to be involved in co-producing healthcare at a national level [4].

Co-design in healthcare involves the equal partnership of individuals who work within the system (healthcare staff), individuals who have lived experience of using the system (patients and their families/carers) and the ‘designers’ of the new system (whether that be IT personnel in terms of electronic platforms to improve efficiency or researchers in terms of designing interventions to improve health systems). Co-design involves working together to design a new product, making full use of each other’s knowledge, resources and contributions, to achieve better outcomes or improved efficiency [5].

In the healthcare literature there are various terms used for different types of co-design activity including experience based co-design (EBCD) which focuses on the use of stories and storytelling by patients to gain a deep appreciative understanding of the strengths and weaknesses of a present service [6], co-production which involves producing a product or service together and comes after the co-design phase [7] and co-creation which usually refers to both co-design and co-production taken together [8].

In this study a collaborative approach to co-design by healthcare staff, patient representatives and advocates, and health systems researchers was adopted with the aim of co-designing a collective leadership intervention to improve healthcare team performance and patient safety culture. This type of co-design is still a relatively novel concept in healthcare and there is a gap in the peer-reviewed literature on *how* to do this type of co-design in practice. This paper addresses this gap by delineating the approach taken in the co-design of this intervention.

### Context of the Co-Design Approach

Traditional hierarchical leadership styles in healthcare are being challenged from a patient safety perspective [9,10] and there is a growing interest in shared or collective leadership styles, which may be defined as “an emergent and dynamic team phenomenon whereby leadership roles and influences are distributed among team members” [11]. Such approaches are characterised by distributed roles and responsibilities and the selective utilisation of the skills and expertise of individuals, as required by the task or situation at hand [12]. Recent research indicates that, across sectors, where shared leadership already exists in teams it predicts team effectiveness [11,13].

The majority of education and development programmes for leaders however focus on developing the individual as an autonomous leader. As it is becoming increasingly evident that the interdependencies in healthcare require more collective leadership approaches [11,13,14,15] there is a need to question the common practice of providing leadership training to a designated leader in isolation from his/her team as well as reconsidering the content, teaching methods, and learning outcomes of leadership programmes. While evidence exists that collectivistic leadership is associated with more effective team performance there is little research to guide us on how to introduce collective leadership to healthcare teams.

This study is part of a wider research programme on Collective Leadership and Safety Culture (Co-Lead) which aims to introduce collective leadership to healthcare teams across one hospital group to improve team performance and patient safety culture [16]. In contrast to traditional approaches that focus development on the individual as leader, the approach in this programme is on developing the team as a dynamic leadership entity, ensuring all members understand and develop the capability for leadership. Rather than starting from a top-down competency framework-driven training programme targeted at the individual as leader, development is being informed through a bottom-up service needs driven co-designed intervention targeted at team members as co-leaders. This represents a radical shift in programme design and delivery and we believe that by involving healthcare team members and patients in co-designing a collective leadership intervention for teams, we will not only design a more authentic and real world solution but also increase ownership of the intervention and improve the likelihood of successful implementation.

In this paper we outline in detail the co-design processes employed in developing a collective leadership intervention for healthcare teams to improve team performance and patient safety culture. Through this exemplar of co-design in practice, we demonstrate the value and potential impact of the co-design approach in helping to inform health system research priorities, intervention design, intervention adaptation and implementation.

## 2. Methods

### 2.1. Ethics

Ethical approval for the study was granted from University College Dublin Research Ethics Committee (ref: HREC-LS-16–116 397/LS-16-20). Local approval was also sought and obtained from the healthcare organisations ethics committees and/or management teams of sites engaged in the research.

### 2.2. Study Context

This study took place with healthcare teams across a newly formed hospital group. It is a large and complex group comprising 11 hospitals working with four Community Healthcare Organisation (CHO) partners and an academic partner. The hospital group serves over one million people.

### 2.3. Establishing a Co-Design Team and Member Recruitment

Three healthcare teams were initially identified by the hospital group senior management to participate in the co-design, implementation and testing of the co-designed intervention (i.e., the Co-Lead intervention). Two further teams were identified during the course of the workshops, based on emerging priorities of the hospital group, and these teams were invited to participate in the co-design process from the third workshop. These include three hospital teams (Acute Medicine Unit, Surgical Ward and an Orthopaedic Team), one clinical academic directorate for cancer care that crosses two hospitals and a university, and an integrated care team for older persons that crosses one hospital and a CHO.

Presentations were made to each team outlining the research programme and aims and a briefing document titled ‘Role Description and Person Specification: Co-Design Team Member (Co-Lead)’ was circulated to senior managers/clinicians on the teams to send to their colleagues to encourage two volunteers from each team to be part of the co-design team. This specification document outlined the level of commitment that would be required (reading and preparing material in advance, attending six three-hour workshops over the course of six months) and the type of person that we were seeking to be part of the co-design team. This included the following:

- Minimum of two years experiences working in healthcare.
- Working in healthcare setting and member of at least one healthcare team consistently for more than twelve months.
- Willingness to work within co-design team to meet shared goals.
- Willingness to listen to, and consider, different perspectives and opinions.
- Good verbal communication skills.
- Commitment to prepare for meetings by reading information sent in advance.
- Interest and enthusiasm in developing effective teamwork and leadership strategies.
- Willingness to share and reflect on personal experiences of team working and leadership (positives and negatives).
- Support and approval from line manager to attend workshops.

Ten healthcare staff volunteered to participate in the co-design team from the five teams identified. Three teams had two volunteers, one team had three and one team had one. They came from diverse backgrounds e.g., nursing, medical, health and social care professionals and brought a wealth of skills, knowledge and experience of being on different healthcare teams. We also invited a patient representative recruited from one of the hospital’s patient liaison service and a patient advocate recruited through Patients for Patient Safety Ireland (PfPSI). Two additional healthcare professionals from the wider healthcare system who were experts in Quality and Safety (Q&S) were also invited to join the co-design team.

Because one patient advocate could not attend due to illness, we also held an additional workshop with two patient representatives who were recruited from research projects the Health Systems team were engaged in and two patient advocates who were recruited through PfPSI. At this workshop, the patient representative who did attend the workshops (AE) jointly presented with the researchers on the process to date and the outputs and facilitated a discussion on further suggestions and inputs.

The researchers on the co-design team were members of the Health Systems research group and included researchers working across a broad range of health and social sciences disciplines including; Psychology, Organisational Behaviour, Organisational Change and Development, Implementation Science, Human Factors/Ergonomics. Seven members of the research team were involved in the co-design process. The full list of participants, their background and experience is presented in Table 1.

### 2.4. Steps in the Co-Design Process

**Figure 1:**
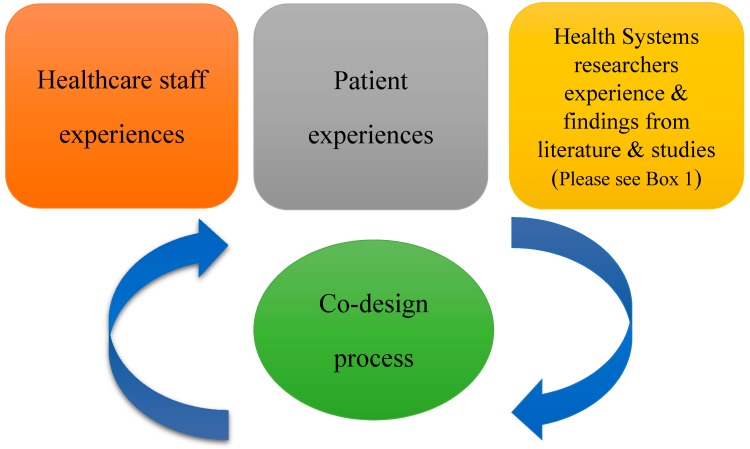
Participants and experiences in the co-design process.

From the beginning of the process we wanted to adhere to both co-design and collective/shared leadership principles and best practices. Thus throughout the course of the workshops there was a symbiotic relationship between the health systems research team and the healthcare staff and patient representative. This is outlined in Figure 1. The researchers delivered short inputs on background, reviews of literature or synthesis of evidence on a topic in response to the needs and priorities that emerged from the co-design discussions (Box 1). Healthcare team members delivered inputs on different aspects of their work, the developing intervention or their experience of the co-design process. The patient representative delivered inputs both as a service user giving his perspective on his experience of team working in healthcare and as someone who has been on a number of different work teams throughout his working career. All team members could set the agenda and prioritise issues/topics for the next workshops.

Box 1 **Evidence reviews and synthesis undertaken by research team to inform discussions during co-design workshops.**

The health systems research team worked on gathering data from extant literature in the following ways:

- A systematic review to explore interventions to develop collectivistic approaches to leadership in healthcare settings
- a narrative review of safety culture in healthcare teams
- a systematic search of the literature with a realist lens on team interventions; what works for whom, in what context and why
- a study on understanding the enablers of nationally identified effective teams
- a study on understanding quality and safety performance measurement and monitoring at the healthcare team level

### 2.5. Structure of the Workshops

Each three-hour workshop began with introductions if new members had joined and icebreaker activities related to the topic of the workshop. These included, for example, discussion in pairs and word association exercises. Throughout the workshops interactive exercises were used to facilitate the co-design process including word associations, questions for small groups, discussion in pairs, personal reflections, developing and discussing case studies, PowerPoint templates to fill in, mapping exercises of possible intervention components, mapping exercises of desired behavioural changes, developing draft implementation road maps, choosing and agreeing evaluation measures and scales.

From the second workshop each one began with a summary of the outputs of the previous workshop and a representation of ‘where we are at now’ in terms of designing the intervention. Workshops were then broken into discrete activities, which provided a chance for all team members to share their experiences of various aspects of team working, collective leadership and patient safety. The researchers would give inputs (e.g., brief presentations or facilitated discussions) based on their previous experience of change management and implementation science in healthcare and the findings from the various literature reviews that were on-going. Each workshop contained a specific ‘co-design’ piece where all team members would actively work on a specific component of the intervention. 

During the second workshop exercises took place on exploring the reality of team working and the challenges that can be encountered and how to overcome these challenges. It was conveyed that it was a difficult topic and people shared some personal reflections about difficulties in team working they had experienced in the past, breaches of trust they had suffered and how they impacted their work environment. The case studies introduced by the research team then helped to bring the discussion back to what might work in their context.

At the third workshop four new members joined the team so we began by presenting the objectives of the overall research programme, what co-design is, and presented an image that included all the possible intervention pieces colour coded according to the three main concepts—collective leadership, team performance and safety culture. Then we moved into exploring the nature of healthcare teams and the complexity of them.

During workshop four we attempted to map the intervention components onto behaviours of team members that we would like to see a change in. This helped to make concrete what exactly we were trying to accomplish with the intervention and it led to rich and detailed discussions on the nature of healthcare teams and the complex environment within which they work. The presentation by one of the healthcare teams at the fifth workshop on their suggested ‘Co-Lead implementation roadmap’ helped team members to get a sense of how what we were co-designing would work in practice. At the sixth and last workshop we discussed in detail the implementation and evaluation of the co-designed intervention. The team decided on evaluation measures and scales.

Each workshop ended with a written evaluation, which included the questions listed in Box 2.

Box 2 **List of evaluation questions for participants following each workshop.**

- Was the workshop worth attending?
- Do you think we are making progress?
- Do you understand how the components of today’s workshop fit with the co-design process?
- Was there any aspect of today’s workshop that worked well/did not work well?
- What is your key take home message?
- Any other comments?

A written summary of the outputs of each workshop was emailed to all team members along with the suggested agenda for the next workshop. Team members were encouraged to review and give feedback on both documents. Appendix A summarises each workshop; the inputs delivered, and the activities carried out within each workshop.

## 3. Results

Through this co-design process, the co-design team members designed a ‘toolkit’ of one-hour interventions to introduce Collective Leadership to healthcare teams with the aim of improving Safety Culture. The intervention sessions would be delivered on a monthly basis and be facilitated by members of a specially established ‘Co-Lead Local Implementation Team’ which would include people who had participated in the co-design process. There are four ‘foundational’ pieces that each team would need to complete. These include:

1. Exploring the team values and establishing a vision and mission for the team
2. Developing role clarity among team members and setting goals for the Co-Lead implementation
3. Equipping the team with skills to discuss patient safety in an open and safe manner
4. Developing a set of meaningful key, safety and quality indicators at the team level to help them monitor their progress

After each team had completed these four interventions a review would take place and the team could select, based on their perceived need and feedback to them from the UCD researchers on the pre-implementation evaluation results, four or more intervention components from the list in Table 2.

This intervention is currently being implemented and tested with four healthcare teams across one hospital group. In this section and for the purposes of this methods paper, reflections on the co-design approach adopted, and the methodological and process considerations we believe were crucial to the success of the practice are presented.

### 3.1. Setting Ground Rules and Structure

#### 3.1.1. Informality, Confidentiality and Timings of the Workshops

Each workshop began with an icebreaker/introductions where each person was encouraged to speak. This helped to put people at ease and set the informal tone for the workshops. Given that we were discussing issues relating to patient safety, improving patient safety culture, team working and examples of good and bad experiences of working on teams, we also asked members if they were happy to keep confidential any sensitive material discussed. At the same time each person was encouraged to share as much or as little as they were comfortable with. This helped to set a tone where people could be honest in their sharing. The workshops took place every month and the dates were scheduled in advance for all the workshops over the six-month period to facilitate planning needed to attend. The workshops were three hours in duration and each one was planned out by the research team, taking on board the input and suggestions, from the co-design team members, at the previous one.

#### 3.1.2. Expertise of the Healthcare Staff

Each person brought a wealth of experience to the co-design process from both their personal and professional lives. The mix of business, nursing, medical and Health and Social Care Professionals (HSCPs) added great depth to the discussion on healthcare team working, collective leadership and safety culture from various perspectives. The healthcare staff noted that for them it was a chance to take time out and reflect on not just their work but *how* they worked and particularly how they worked and interacted with others. For some, it was the first opportunity they had to do this.

#### 3.1.3. Role of the Patient Representative

A lot of emphasis has been placed in recent years on researchers involving patients and their families/carers and members of the public in research to improve health systems. We found the input of our patient representative throughout the six workshops to be invaluable. They brought the rich experience of their perception of how healthcare teams worked, communicated with each other, and their impressions of relationships among healthcare team members. They helped the whole team to keep focused on the ultimate goal of the research programme, which is to improve patient safety culture. The additional workshop with the patient representatives and patient advocates was very informative and helped shape our definition of healthcare teams to be more inclusive of all staff who interact with and help meet patients needs on the ward from catering, cleaning, portering staff, health care assistants, nursing, medical and health and social care professionals.

#### 3.1.4. Inputs from the Research Team and the Q&S Experts

The research team fed into the co-design process from their backgrounds and experience but also from the literature reviews and studies of existing practice. These inputs were delivered both on a planned, structured basis but also in response to the topics that were emerging from the team. When the team members discussed or felt an issue was important in terms of team performance, this was researched and findings fed back at the next or subsequent workshops. The whole process of co-design involved an on going dialogue between the healthcare staff, patient representatives and the researchers, which included periods of listening, reflecting and speaking. The Q&S experts played an invaluable role in ensuring that we were not ‘reinventing the wheel’ with any aspects of the intervention and directing us towards tools and initiatives that were already in place in healthcare to improve team functioning and safety culture for example the Agency for Healthcare Research and Quality (AHRQ) Team Strategies and Tools to Enhance Performance and Patient Safety (TeamSTEPPS).

#### 3.1.5. Exercises and Discussions

For each workshop exercises were given either in advance or during the workshops to stimulate discussion on topics or more in-depth analysis of different components of the intervention and how they might work. Workshops were very interactive with lots of different types of activities to maintain interest throughout. Individual reflections and exercises took place along with discussions in pairs, small groups and the larger group.

#### 3.1.6. Evaluation Process

At the end of each workshop a short written evaluation took place, which included the question listed above in Box 2. The results were reflected on between workshops and helped create the agenda for the next one. At the last workshop an evaluation took place of the process across the six workshops. A formal evaluation of team members’ experiences of the co-design process is on-going with researchers who were not involved in the co-design process but we draw on aspects of the written evaluations here to help illuminate the process.

### 3.2. The Co-Design Process

The process of co-design involved bringing people together who would not normally come together to co-design a collective leadership intervention. There was little guidance in the literature on both how to do this type of co-design and on how to develop collective leadership in teams. Our aim was to design an intervention that was grounded in the real-world experience of healthcare staff and in their contextual reality through a co-design process, whereby all co-design team members have an equal voice and role in prioritising and designing content. Thus, we began with a broad aim but with no preconception of what a final intervention might include or look like. We began the co-design process by exploring the reality of working in a modern healthcare team to help ensure the intervention was designed to be grounded and feasible given those realities. Our five teams represented in the co-design process different healthcare team types and therefore we spent time exploring these differences and learning about the context within which each team operated. Members of the research team had different levels of awareness of collective leadership, effective team performance and patient safety culture. While we brought this to the table with us we attempted at all times not to influence discussions but rather to allow topics emerge from the exercises and activities the whole co-design team engaged in. From the evaluation of the first workshop people noted there was great enthusiasm for the process, they felt very ‘engaged’, felt there was good momentum during the workshop and that they had a good grounding in the core concepts. People also commented on the make up of the team e.g., ‘Great diversity in the room and wealth of experience. Will be great to harness it’.

Following the second workshop, the evaluations reflected the challenges discussed in relation to trying to change how people work and one person noted that their key take home message was the ‘Complexity and challenges of change, need to return to the fundamentals and put the ‘human’ component back in team-working and patient care’. The evaluations from the third workshop were positive but they also reflected a growing awareness of what co-design is (‘Am beginning to think the workshop will inform the design as opposed to producing the end product’) and the difficulties in taking everyone’s perspective on board (‘Difficult to balance allowing people have their say whilst not getting entrenched on one idea. Different people at different stages in terms of their thinking. This is reflective of the need to take into account real world healthcare for intervention’).

Following the fourth workshop there was a sense of frustration that we were not making progress (‘I feel we are in danger of talking ourselves to death about the same subjects, interesting and all as they are!) and that the reality of what we were trying to co-design would not be implementable (‘Resources [for healthcare teams] are an issue. Protected time has been highlighted repeatedly, therefore the intervention has to be simple and easily accessible and implementable’).

The presentation by one of the healthcare teams at the fifth workshop on their ‘roadmap’ helped team members to get a sense of how what we were co-designing would work in practice. Team members noted that there were a ‘lot of breakthrough’ moments at this workshop and there was a sense of things ‘coming together’ (‘Feels like we’re making great progress and intervention becoming clearer’, ‘We can make a real difference with some manageable interventions’). At the sixth and last workshop the evaluations reflected a sense of things ‘coming together’ but that still a lot of ‘detail’ needed to be worked out.

On the overall evaluation of the co-design process people felt that it was frustrating at times and ‘starting from a blank canvas has meant it has been slow at times’. Other people felt that despite our best efforts to remain open and allow the intervention to emerge at times we still slipped back into old ways of ‘prescribing’ how things should be done. People however did see the value in ‘the diversity between the teams’ and acknowledged that this led to a ‘fuller understanding of what is required and that there will be different interventions required’. Finally one person noted that co-design is all about being ‘comfortable with the uncomfortable’ and that we had made a lot of progress from a ‘blank page’.

## 4. Discussion

Through the co-design process a collective leadership intervention was developed for healthcare teams, by healthcare staff, researchers and patient representatives and advocates, taking account of the complexities of the operational reality of healthcare. This intervention represents a radical departure in leadership capacity development in healthcare and we believe it has created a rich suite of interventions that will enable collective leadership in healthcare teams and improve safety culture. The intervention is currently being piloted with four teams across one hospital group.

### 4.1. Benefits of Taking a Co-Design Approach

One of the clear benefits of co-design that emerged in this study was beginning the design process from the reality of people’s everyday work environment rather than designing from theory something that ‘should’ work for them. Having patient representatives and advocates in the room doing this added another dimension to the process by helping to keep in mind at all times the ultimate aim of improving the healthcare system. Other studies have spoken of the ‘considerable depth and richness’ that emerges through the co-design process [2] and in particular through the involvement of patient representatives and advocates [17]. In this study remaining open to the co-design process enabled the development of a multi-level collective leadership intervention that is cognisant of the need to build the ‘foundations’ of building personal relationships, building trust, creating a safe space to talk openly about safety etc. Our understanding of healthcare teams has changed as a result of this process and has led us to adopt a very open definition of a modern healthcare team as (a) composed of two or more individuals, (b) who exist to perform organisationally relevant tasks, (c) share one or more common goals, (d) interact socially, (e) exhibit task interdependencies (i.e., workflow, goals, outcomes), (f) maintain and manage boundaries, and (g) are embedded in an organisational context that sets boundaries, constrains the team, and influences exchanges with other units in the broader entity [18]. Similarly our understanding of what collective leadership would look like in practice has changed and led us to define it simply as allowing each team member to contribute to the working of the team in ways that allows them to use their skills and talents to the optimum.

Another benefit of taking a co-design approach for us was that the team members who participated in the co-design process and are now implementing the Co-Lead toolkit and the philosophy of collective leadership with their local healthcare teams. Research from implementation science, health services delivery and quality improvement literature consistently highlights that quality improvement results often fall short of expectations and promising interventions shown to be initially successful are often hampered by sustainability issues. Estimates indicate that healthcare organisations experience implementation failure in almost two-thirds of initiatives manifesting in the ‘quality chasm’ [19]. Therefore, even interventions with proven effectiveness fail to translate into meaningful patient care outcomes. The NHS refer to this as the ‘improvement-evaporation effect’ [20,21]. There is substantive research that has identified the diverse range of contextual factors that can impact on the success and sustainability of quality improvement efforts [22,23]. The construct of mutual adaption and co-creation of both programme and context is considered critical to achieving sustainability [23,24]. Co-designing, co-producing and co-creating interventions at the beginning of research programmes rather than when trying to sustain interventions that may not have been suited to the environment in the first instance, may lead to more success in improving healthcare. It is our intention to evaluate the impact of having staff who were involved in the co-design process involved in the process of implementing the toolkit. From our early experiences of implementation we would recommend co-design for healthcare team based quality and safety improvement interventions and in particular where team members have an opportunity to be involved in the co-design process.

### 4.2. Challenges of Taking a Co-Design Approach

Co-design however is not an easy methodology. It involves a letting go of *a priori* assumptions, in this instance about healthcare teams and settings, about what collective leadership would look like in practice, about what healthcare teams need to improve their performance and about their understanding of safety culture and how to change it. Co-design is a labour intensive process and involves *real* listening to the ‘different’ perspectives in the room and attempting to understand each person’s reality as different but complementary to others. It takes a lot of time to prepare and facilitate sessions and requires facilitators to always have the bigger picture in mind. At times it can be tempting to fall back to prescribing ‘these are the steps in implementing change’ or ‘these are the components to develop collective leadership’. It involves a challenge to move out of one’s ‘comfort zone’ [17]. Co-design also presents challenges to researchers from an ethics and grant writing perspective when, for example, outcome measures cannot be pre-specified [25].

### 4.3. Limitations of This Study

Some limitations of this study are that due to work pressures only two teams managed to have two representatives on a regular basis and two teams only had one representative at any time. One of the patient advocates did not make any meetings due to illness. Maher et al. [26] similarly found that patient attrition is a factor in co-design and patient availability needs to be considered early. The likelihood that patients may not be able to continue their input should be anticipated. They recommend engaging with a number of patients from the start and plan on going recruitment. To gain further patient representative and advocates feedback we held an additional workshop with a group of five invited patient representatives and advocates. Also while the evaluations used at the end of each workshop were paper based and could be completed anonymously if people wished, there still may have been some social desirability influences in the results. The co-design process should ideally be evaluated by researchers independent of the process. The development and implementation of the co-designed intervention is currently being evaluated by researchers who were not involved in the co-design workshops.

## 5. Conclusions

Taking a co-design approach to developing the collective leadership intervention in our research programme was extremely beneficial in informing the development of the intervention and the different components of the Co-Lead toolkit. Through harnessing the collective expertise of all team members the co-design process also encouraged us to consider feasibility of implementation throughout the design period as well as the appropriateness of possible intervention components to the local contexts of the teams. Not only was the intervention informed by the knowledge and experience of each co-design team member but also through the process of co-design each of the members developed a greater awareness and appreciation for the intervention components and therefore there is greater ownership and engagement by the healthcare teams where the intervention is currently being implemented. Co-design has great potential as a methodology for understanding health systems and for enhancing research outputs and potentially, implementation success.

## Figures and Tables

**Table 1:** Co-design team members.

Background	Intervention Team/Area of Expertise
Staff Nurse	Acute Medicine Unit
Doctor (Registrar)	Acute Medicine Unit
Assistant Professor in Nursing/Researcher in Cancer Care	Cross centre cancer team Practicing Nurse—honorary clinical role
Consultant Medical Oncologist	Cross centre cancer team
Assistant Director of Nursing	Surgical Ward, Perioperative Directorate
Business Manager	Surgical Ward, Perioperative Directorate
Clinical Specialist Physiotherapist	Orthopaedic Team
Care Co-ordinator	Community Healthcare Organisation
Senior Occupational therapist	Community Healthcare Organisation
Senior Physiotherapist	Community Healthcare Organisation
Consultant Paediatrician	National Clinical Lead Quality and Safety and practicing consultant
Renal Consultant	Consultant & Clinical Lecturer, Risk and Change Management, Medical Ethics
Patient Representative	Identified by hospital patient liaison officer
Patient Advocate	Patients for Patient Safety Ireland (PfPSI)
Prof. of Health Systems	Psychology, Organisational Development, Organisational Behaviour
Senior Research Fellow in Health Systems	Psychology, Organisational Behaviour, Human Factors/Ergonomics
Research Fellow	Psychology, Organisational Behaviour, Implementation Science
Strategy Development Officer	Healthcare team effectiveness, organisational strategies to promote team effectiveness, medial law
Project Manager	Research project manager
Research Assistant	Psychology, psychological safety
PhD Student & hospital manager	Head of Transformation Office, Chair of Health & Social Care Professionals Directorate

**Table 2:** List of optional team intervention components.

Collective Leadership for Team Performance	Collective Leadership for Safety Culture
Effective Team Meetings Business Case for Improvements Removing Frustrations/Pebbles in our shoes Improving Communication Inter-disciplinary Ward Rounds Person-centred work environment Joy & meaning at & in work Emotional Support for the team Emotional Support for the individual following incident Team Status Review	Building Trust within the Team Challenging Unsafe Behaviours (from TeamSTEPPS) Collective Leadership for Safety Skills Understanding Safety Performance at the Team level Situation Awareness Communication at Safety Critical Moments: Shift Handover Communication at Safety Critical Moments: Patient Care Handover Safety Walk Rounds at the team level High Reliability/Collective Mindfulness at the Team level Sustaining Improvements

## Appendix A

**Table A1:** Workshop Inputs, Exercises and Outputs.

WS	Inputs	Exercises
WS1 January 2017 3 h workshop	Healthcare Team members: Presentation by team members on work of their team Health Systems Researchers: Presentation on Co-Lead research programme Presentation on Co-design as a method and the aims of the intervention and how we will evaluate it including exploring how teams currently measure team performance	Introductions Word association exercise using words: effective teams, patient safety, safety culture, leading in teams, joy and meaning Small group exercise: How can teams know they are performing well? What helps teams to know they are performing? How can teams consider patient safety? How can teams know they are contributing to the achievement of organisational goals? What values should guide healthcare teams? Discussion in pairs: Suggestions for planning components of future workshops
WS2 February 2017 3 h workshop	Healthcare Team members Reflection Exercise given in advance: Please reflect on a time when you were a member of a team that did not function so well, where being on the team was a source of frustration for you. Number of question given to aid reflection. Please read the following article: Weller J, Boyd M, Cumin D. Teams, tribes and patient safety; overcoming barriers to effective teamwork in healthcare. Postgrad Med J 2014; 90:149–154. Health Systems Researchers Presentation on trust as trust came up a lot in first session as essential for team performance.	Feedback from all Co-Design team members on Reflection Exercise People were asked to divide into pairs, share as much as comfortable from personal reflection on experience of being in a team that did not function so well. Individual feedback was gathered through Stickies exercise on 5 levels of barriers identified in reflection exercise Feedback was invited in main group on 5 levels and also on any learning from Weller article on how to overcome any of the barriers that might have been raised here. Developing Trust in healthcare teams Individual word association exercise before presentation and group discussion after. One different Case Study of effective team interventions given to three small groups to discuss following question in relation to each: Why do you think the intervention worked? Do you think this intervention would work in your organisation/team? What aspect do you think could be incorporated into the co-design intervention?
WS3 March 2017 3 h workshop	Healthcare Team members Each of the teams represented were given a PowerPoint template to fill in details on their team around the following themes: How the team was formed; team membership; understanding of roles and responsibilities on the team; communication, co-ordination and collaboration across team members; leadership and decision-making; dignity, social support and trust; relationship with the wider hospital, hospital group, health service. Health Systems Researchers Presented ‘honeycomb’ format of possible intervention pieces colour coded according to the three main concepts—Collective Leadership, Team performance and Safety Culture.	Each team presented on their ‘homework’ by using the PowerPoint template. Health Systems Researchers Exercises on developing intervention to improve team roles and relationships
WS4 April 2017 3 h workshop	Healthcare Team members Teams were given the exercise of developing an induction pack or set of headings for an induction pack to induct new people onto the team Health Systems Researchers Bringing together all the work to date from the first three workshops. Presented revised ‘honeycomb’ format of possible intervention pieces colour coded according to the three main concepts—Collective Leadership, Team performance and Safety Culture. Mapping the relationship of Co-Lead concepts (Collective Leadership, Team Performance and Safety Culture) onto Behaviours we hope to impact onto Intervention Components to change those behaviours Developing Collective Leadership Components of the Intervention	Presentation of draft and discussion on the three main concepts and how they relate to team behaviours and to the possible components of the intervention So for example: What behaviours would be witnessed / evident in a team where there was Collective Leadership? List all behaviours What intervention(s) would foster this / these behaviours? List all interventions according to the Collective Leadership behaviour they would promote. Taking a few Collective Leadership interventions and outlining those and asking the Co-design team to discuss if they would work in their team/organisation. It was felt we had covered some Team Performance interventions already but had not focused on Collective Leadership. Safety Culture it was felt could come later. “if you wanted to improve CL on your team, what would you do with your team? How would you do it and when would you do it?”
WS5 May 2017 3 h workshop	Healthcare Team members Two teams asked to think, based on the development of the intervention to date, which parts and how they would go about implementing it with their team. They were asked to give a presentation on this at the next workshop. National Clinical Lead Q&S made a presentation on Safety Culture and different frameworks to explore patient safety, safety culture, safety measurement. Health Systems Researchers Presentation on the behaviours needed for a good Safety Culture and what types of intervention would help foster those. Measurement and Monitoring of Patient Safety framework (Vincent et al. 2013) & results of study into what data is being gathered at the team level.	One team presented a ‘roadmap’ of a possible suite of interventions they felt their team needed based on the honeycomb map. Exercise on co-designing team-level Q&S indicators “What would you like to know about team performance?” The overall structure/template for the draft intervention toolkit was presented and some sample tools. Discussion on this. Two interventions were discussed in more details: Role Clarity and Team Charter/Team induction
WS6 June 2017 Day long workshop	Healthcare Team members Presentation on current status of Collective Leadership for Safety Culture Intervention Toolkit which included a set of ‘Foundational Components’ that each team should do and then a set of ‘Targeted Components’ that teams could pick and chose from depending on their needs and what emerged from the initial implementation of the Foundational Components. Facilitated discussion on Implementing the Intervention and the practicalities involved. Presentation on the suggested evaluation approach and scales and how each team would also select their own QPI/SPI to review and facilitated discussion on this. A discussion took place on the involvement of patient representatives in the process and how only one patient representative made the workshops. A spoken and written evaluation was carried out. The co-design team were reminded of each workshop’s activities and asked to evaluate their experience of the overall process.	Each person was asked to review: what would work for your team from current intervention components what is still needed for your team based on current list of component pieces The practicalities of implementing the intervention were discussed in detail: identifying intervention teams identifying local team members to run with the intervention involving PPI on each intervention team exploring what interventions have taken place with this team before identifying barriers and facilitators identifying start date for rolling out intervention setting up meetings with each intervention team to begin process/hospital senior management to inform of process Exercise to decide on scales to measure Effective team performance Collective leadership Safety culture Discussion on how to get further feedback from Patients, Patient Reps, Public on Toolkit
Workshop with Patient Reps & Advocates July 2017	A joint presentation was made by members of the research team and the patient representative who had participated in the co-design process on both the process of designing the intervention and the intervention components that had been agreed on along with evaluation criteria.	Small group discussions on their experiences of healthcare teams in hospitals or other healthcare settings and how that team’s functioning could have been improved from a patient safety perspective (to begin identifying any gaps in the intervention toolkit). Small group discussion following presentation on Co-Lead on how team’s performance could have been improved from a Collective Leaderhsip perspective (to continue identifying any gaps in the intervention toolkit). Detailed discussion in small groups on the intervention toolkit pieces and any gaps. Group discussion on implementation and evaluation, ideas/suggestions around this and their possible role in both.
